# The evolution of ABC star polymers: from trial-and-error to rational design

**DOI:** 10.1039/d5ra03730a

**Published:** 2025-08-04

**Authors:** Matus Kalina, Babak Nouri, Kristoffer Almdal

**Affiliations:** a Department of Chemistry, Technical University of Denmark Kgs. Lyngby Denmark kral@dtu.dk

## Abstract

ABC star polymers, consisting of three chemically distinct polymeric chains bound to a common point, have emerged over the last 35 years as versatile materials with tunable morphologies and potential applications in nanofabrication, drug delivery, or solid-state electrolytes. Despite decades of progress, well-defined synthesis and design remain a challenge due to their high synthetic complexity. This review surveys key developments in synthetic strategies, ranging from early anionic routes to modern reversible-deactivation radical polymerizations and click-driven methods, highlighting the trade-offs between architectural precision, functional compatibility, and scalability. Particular emphasis is placed on the resulting morphologies in bulk, thin-film, and solution states, where the star topology enables unique structural motifs not accessible to linear triblocks. These include complex tilings, hierarchical phases, and multicompartment micelles. Emerging computational and data-driven approaches are discussed in the context of inverse design, offering new directions for bridging idealized model systems with scalable, application-ready materials.

## Abstract 2

Three incompatible polymer chains meet at a single junction in ABC star polymers, forming a confined architecture that drives unusual structural behavior and underpins their relevance in polymer science.

## Graphical abstract

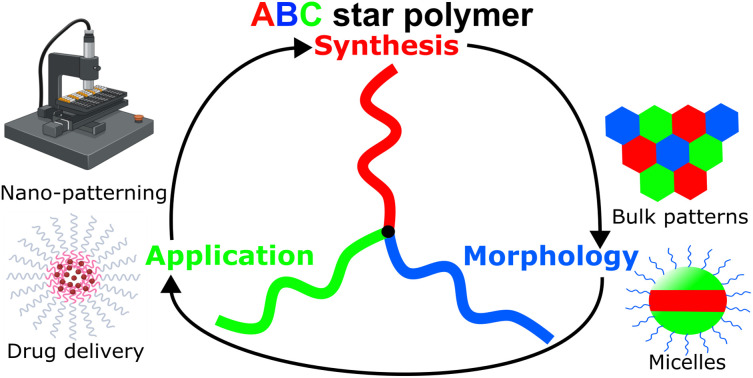

## 1 Introduction

Polymers with controlled architectures have long been pursued for their tunable material properties, enabling precise control over performance. Even with identical chemical composition, the arrangement of chains, whether linear, branched, or cross-linked, can determine a wide range of physical properties, from thermal and solubility behavior to conductivity,^1–5^ as well as nanoscale structure and morphology.

One such innovation is the ABC miktoarm (µ-star) polymer, named from the Greek miktos, “*mixed*”, in which three chemically distinct polymer arms are bound to a central junction. These will be referred to as ABC stars throughout this review. While conceptually elegant, their synthesis presents unique challenges. Unlike linear block copolymers (BCPs) or symmetric star polymers, ABC stars require precise control to link three distinct arms at a single defined junction, demanding high reagent purity and stringent exclusion of side reactions.^6–9^

Over time, synthetic strategies evolved to address these challenges, notably through the use of heterofunctional initiators that enabled orthogonal polymerizations to proceed directly from multifunctional core molecules.^10^ The implementation of click chemistry further expanded the coupling possibilities: azide–alkyne cycloadditions, thiol–ene reactions, and even triple-click sequences enabled rapid, modular coupling under mild conditions with minimal purification.^11–13^ Despite these advances, ABC stars remain a benchmark for synthetic precision in macromolecular chemistry.

Beyond their synthetic challenge, ABC stars have long been valued for their distinct self-assembly behavior. The covalent linkage of three chemically incompatible polymer blocks at a single junction imposes strong constraints on chain organization, promoting microphase separation both in solution and in the bulk. A longstanding goal in amphiphilic polymer design has been the creation of multicompartment micelles, in which each block forms a separate domain. ABC stars offer a direct route to such structures, enabling micelles with internal segregation, segmented coronas, and complex anisotropic morphologies.^14,15^ In the bulk, composition-dependent assembly leads to an exceptionally rich palette of nanostructures,^16,17^ including hierarchical domains and periodic tilings not accessible to linear triblocks.

These self-assembled structures have inspired interest in a range of functional applications. In drug delivery, the compartmentalized architecture of ABC stars enables simultaneous encapsulation of hydrophobic and hydrophilic agents, with the potential for selective, stimuli-responsive release.^18^ Their nanoscale ordering and domain tunability also hold promise for nanolithographic applications, where phase-separated morphologies can serve as templates for high-resolution pattern transfer.^19^ In electrochemical systems, the ability to spatially segregate ion-conductive and insulating domains offers routes toward improved solid-state electrolytes.^5^ These emerging applications are closely tied to the interplay between chemical structure and self-assembly, which will be revisited in the later sections of this review.

While other star polymer systems have been reviewed elsewhere,^20,21^ this work focuses specifically on ABC stars, defined by their three chemically distinct arms joined at a single junction. Emphasis is placed on experimentally realized structures, with three main goals: (1) to introduce and contextualize the main synthetic strategies used for ABC star preparation, including anionic polymerization, ring-opening polymerization (ROP), reversible deactivation radical polymerization (RDRP^22^), and modern click-based or modular approaches; (2) to examine how these methods have enabled diverse self-assembled morphologies in the bulk, in solution, and under stimuli-responsive conditions; (3) to briefly highlight the functional applications that arise from the interplay between structure and assembly. By consolidating synthetic tools, design examples, and structure–property relationships, this review aims to serve as both a comprehensive reference and a practical guide for researchers working with or entering the field, particularly those with a background in polymer chemistry or soft materials science.

## 2 Synthetic strategies for ABC star polymers

The synthesis of ABC star polymers builds upon decades of progress in living polymerization^23^ and star-shaped architectures.^24–29^ These advancements resulted in precise multi-arm constructions, eventually culminating in the first ABC stars in the early 1990s.^30,31^

**Fig. 1:**
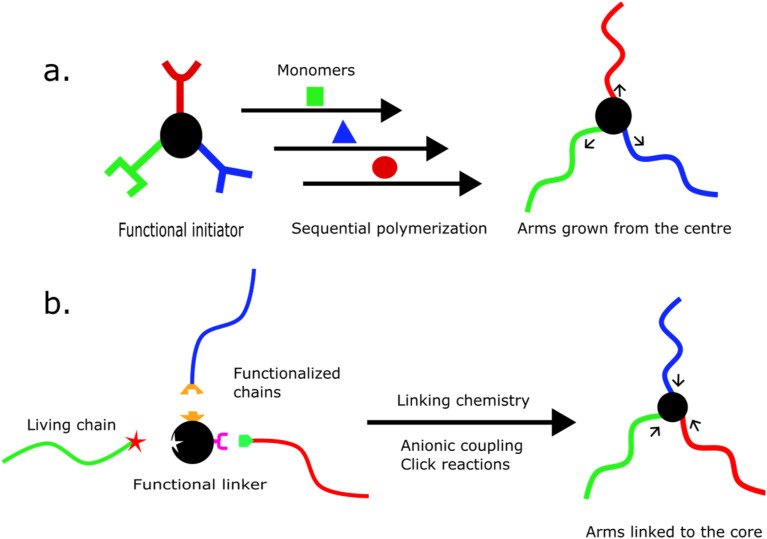
(a) Core and (b) arm-first techniques for preparation of ABC star polymers.^35^

ABC star syntheses are categorized into two principal strategies: the “core-first” and the “arm-first”, illustrated in Fig. 1. In the core-first method, arms are grown sequentially from a multifunctional core *via* orthogonal polymerization techniques, using distinct functional groups embedded in the core molecule. In the arm-first approach, preformed homopolymer chains are coupled to a central junction.^32^ This category includes both anionic and click-based linking strategies, among others, and may resemble grafting-onto methods depending on the chemistry. Although divinyl crosslinkers have played a role in star polymer synthesis,^33,34^ their application in well-defined ABC stars remains limited and is not discussed further.

These strategies rely on different polymerization techniques, each offering trade-offs in scope and precision. The following sections outline how anionic polymerization, RDRP and ROP, combined with linking chemistry, have been used to construct ABC stars.

### 2.1 Polymerization techniques

Anionic polymerization laid the foundation for ABC star synthesis, offering unmatched control over chain length, dispersity, and end-group fidelity.^36^ Initiated by strong nucleophiles, the process proceeds without termination or chain transfer, forming living chains with nearquantitative end-group incorporation. These polymerizations occur *via* carbanionic active centers in nonpolar solvents that suppress side reactions. Rapid initiation and absence of termination yield narrow dispersities,^37^ while counterion identity and solvent polarity influence microstructure.^38^ The monomer scope is largely limited to polystyrene (PS), polyisoprene (PI), polybutadiene (PB), polydimethylsiloxane (PDMS), and selected (meth)acrylates. Solvent compatibility is also limited, favoring aprotic, low-dielectric media to preserve carbanionic activity.^39^ Despite these limitations, anionic techniques remain the benchmark for architectural precision, especially in systems emphasizing structure–morphology relationships.

ROP has been pivotal due to its ability to incorporate cyclic segments such as poly-ε-caprolactone (PCL), poly(ethylene oxide) (PEO), and polylactide (PLA). It operates *via* a ring-opening mechanism, typically through coordination–insertion catalysis,^40^ where nucleophilic initiators, commonly alcohols, open cyclic monomers under metal, or organic-based catalysts.^41^ This mechanism enables chain growth with predictable molecular weights and low dispersity, while maintaining active chain ends for further functionalization.^42^ The compatibility of ROP with a wide range of hydrophilic and biodegradable monomers has made it an attractive route for generating ABC stars with amphiphilic or responsive segments.

Reversible deactivation radical polymerization (RDRP) became widely used in ABC star syntheses due to its tolerance to diverse functional groups and modular end-group design. It enables living-like control *via* reversible deactivation of propagating radicals. In atom transfer radical polymerization (ATRP), this is achieved through halogen exchange with metal catalysts;^43^ Reversible Addition–Fragmentation Chain Transfer (RAFT) uses thiocarbonylthio agents for degenerative chain transfer;^44^ and nitroxide-mediated polymerization (NMP) employs persistent nitroxide capping.^45^

Though RDRP yields broader dispersities than anionic methods, it tolerates monomers incompatible with ionic routes, including acrylamides, hydroxyalkyl acrylates, and zwitterionic or acid-functional vinyl species. Representative segments include poly(*N*-isopropylacrylamide) (PNIPAM), poly(2-hydroxyethyl acrylate) (PHEA), and poly(acrylic acid) (PAA). These often feature protic or highly polar groups that destabilize anionic propagating species. RDRP's compatibility with aqueous and mild conditions makes it ideal for synthesizing responsive, biofunctional, or charged ABC stars. The overview of the techniques, highlighting their advantages and limitations is shown in Table 1.

**Table 1:** Overview of the polymerization techniques used to construct ABC stars

Approach	Advantages	Limitations	Segments	ABC applications
Anionic	Precise *M*_n_, living character, end-group fidelity^46^	Strict anhydrous conditions, limited monomers^47^	PS, PI, PB, PDMS	Well-defined, morphology studies
ROP	Biocompatible segments, low dispersity^42^	Cyclic monomers, metal catalyst residues^48^	PCL, PLA, PEO	Biodegradable, amphiphilic
RDRP	Monomer tolerance, mild conditions^49^	Dispersity, end-group instability^43,44^	PNIPAM, PAA, PHEA	Stimuli-responsive, modular

### 2.2 Linking strategies

ABC star synthesis demands precise and efficient linking of chemically distinct arms at a single junction. As each arm must be incorporated in a defined sequence and orientation, the linking step requires high yield and functional group compatibility to preserve architectural precision.

**Fig. 2:**
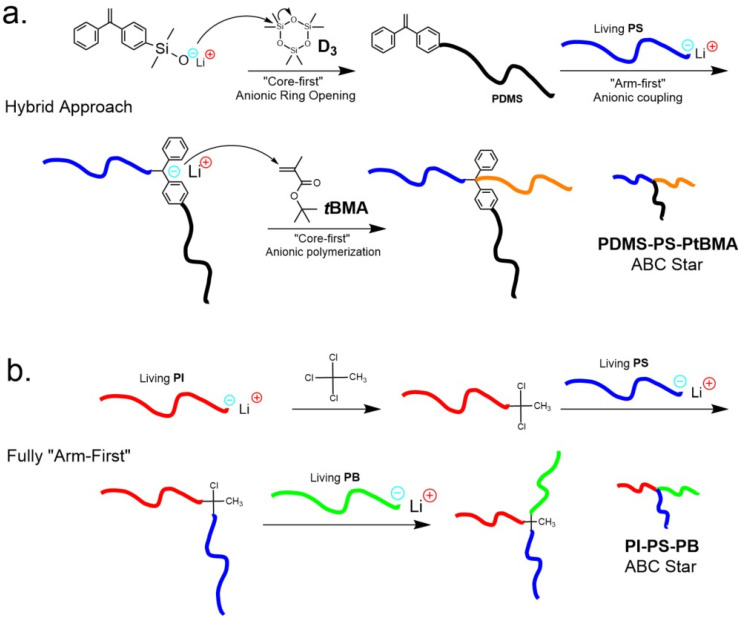
The synthetic pathways of the first two reported ABC stars. (a) DPE-based synthesis by Fujimoto *et al.*,^30^ (b) chlorosilane-based synthesis by Iatrou *et al.*^31^ Adapted from ref. 30 and 31.

In anionic syntheses,^30,31^ living chains are linked to 1,1-diphenylethylene (DPE) or chlorosilane cores under stringent conditions (see Fig. 2). Nucleophilic carbanions attack vinyl or silicon centers, forming robust C–C or Si–C linkages. While highly effective, they remain confined to a rather narrow group of compatible monomers and solvents.

By contrast, ROP is generally used in a core-first fashion, with limited application in post polymerization coupling. However, terminal hydroxyl groups can undergo esterification.^50^ RDRP offers greater flexibility in end-group design. Its broad functional group tolerance allows incorporation of reactive handles such as azides, alkynes, and thiols, enabling modular designs with more readily functionalized small-molecule cores. This has made click chemistry particularly attractive for ABC stars, enabling orthogonal, high-yielding couplings under mild conditions.^51^

Among these, the copper(i)-catalyzed azide–alkyne cycloaddition (CuAAC) forms triazoles through the addition of azides to terminal alkynes;^52^ thiol–ene reactions proceed *via* radical-mediated addition to C=C bonds;^53^ and Diels–Alder (DA) reactions couple dienes with dienophiles *via* thermoreversible [4,2] cycloadditions.^54^ A comparative overview of these strategies is given in Table 2.

**Table 2:** The most common linking strategies in the field of ABC stars

Linking	Components	Notes	Ref.
DPE	Carbanion, DPE	Precise stoichiometry	30, 55 and 56
Chlorosilane	Carbanion, Si–Cl	Demanding conditions	31, 57 and 58
		Long reaction times	
CuAAC	Azide, alkyne	Cu(i) catalyzed, mild, orthogonal	11, 59 and 60
Thiol–ene	Thiol, alkene	Radical-mediated, tolerant to aqueous media	12 and 61
Diels–Alder	Diene, dienophile	Thermally reversible	62–64

ABC star synthesis has evolved through increasingly sophisticated combinations of the above techniques, with priorities shifting from structural precision to degradability, responsiveness, and function. To reflect on these developments, the following discussion is structured into five sections: (1) anionic routes; (2) ROP-based and biodegradable systems; (3) RDRP-based and responsive architectures; (4) distinct examples; (5) applications of ABC stars.

## 3 Synthetic development of ABC star polymers

### 3.1 Carbanions and control: evolution of anionic polymerization

The first ABC stars were synthesized in the early 1990s using anionic techniques. Fujimoto *et al.* linked a living PS chain to a DPE-functionalized PDMS macroinitiator followed by anionic polymerization of *tert*-butylmethacrylate (*t*BMA).^30^ Shortly after, Hadjichristidis *et al.* developed a chlorosilane-based coupling route with a superior control, using methyltrichlorosilane to link PI, PS, and PB arms by exploiting differences in nucleophilicity and steric hindrance.^31^ Both of these foundational approaches are shown in Fig. 2. Bellas *et al.* later addressed the high dispersity of PDMS-containing stars by introducing two-stage temperature-controlled polymerization to suppress backbiting and eliminate fractionation.^58^

Anionic ABC stars gradually incorporated more complex and functional monomers. Methyl methacrylate (MMA) and 2-vinylpyridine (2VP) were early additions, introduced *via* DPE and chlorosilane-based methods to yield PS–PI–PMMA,^57^ PS–PB–PMMA,^65^ PSPB–P2VP^66^ and PS–PI–P2VP^67^ stars with tunable polarity and responsiveness. P2VP, in particular, enabled hierarchical morphologies and metal coordination^68–70^ that became central to bulk morphological studies. 1,3-Cyclohexadiene was later incorporated using PI seeding to overcome low reactivity by Wang *et al.*,^71^ eventually improved by Uhrig *et al.* under nonpolar conditions.^72^

More recent efforts focused on functionality: Hirano *et al.* prepared ferrocene-bearing stars;^73^ Nunns *et al.* introduced ferrocenylsilane blocks *via* CuAAC after chlorosilane attempts failed,^74^ and Higashihara *et al.* developed conjugated systems based on poly(3-hexylthiophene) using both anionic and click-based routes.^55,75^

**Fig. 3:**
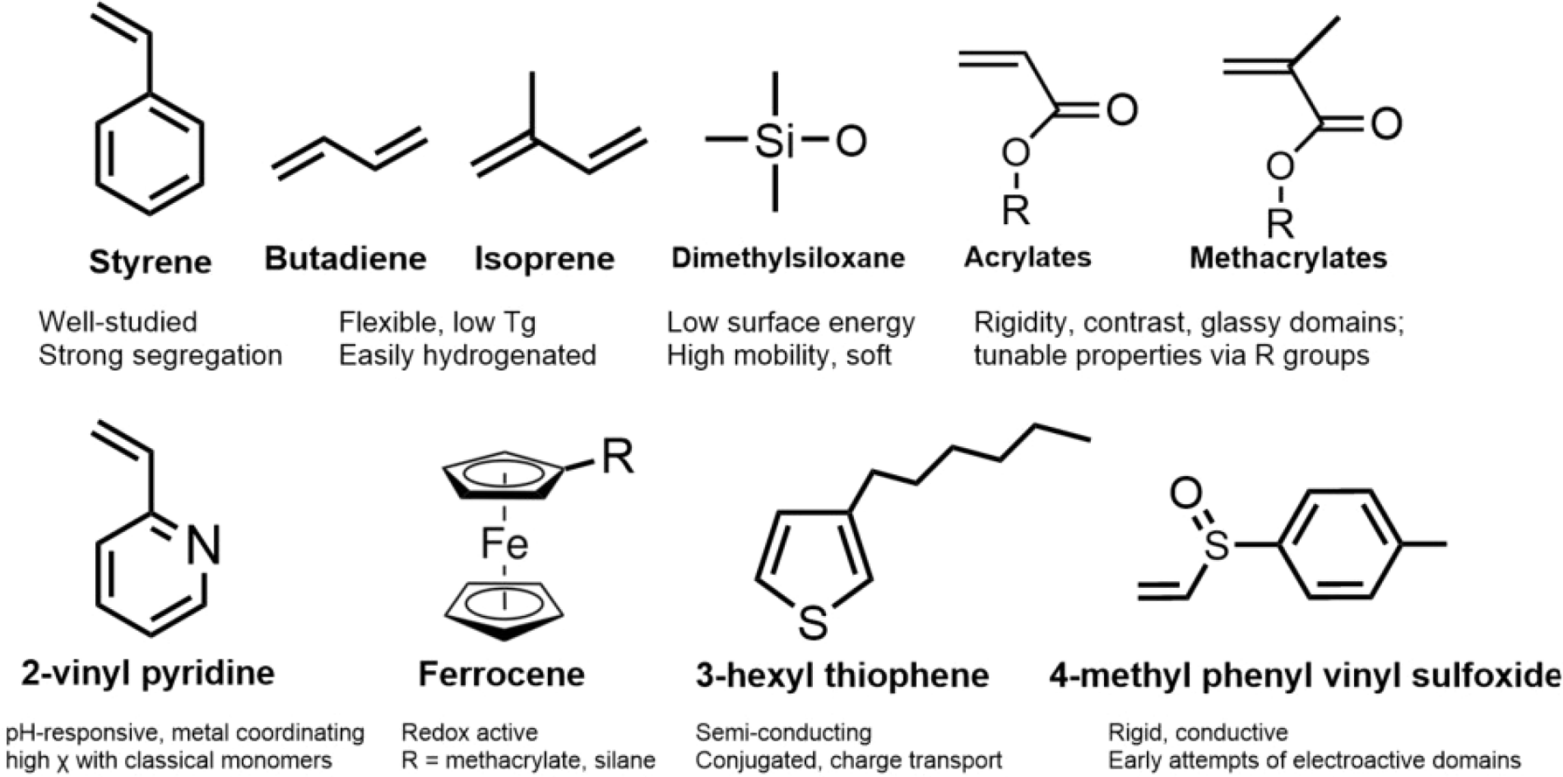
Representative monomers employed in anionic ABC star synthesis.

These advances expanded the synthetic space beyond hydrocarbon monomers, enabling redox-active, conjugated, or metal-coordinating domains with stimuli-responsiveness potential. Incorporating such features into ABC stars allows access to pH-, redox-, or thermally responsive behavior, which can drive changes in conformation, assembly, or solubility, depending on the environment. These properties have become increasingly important for designing adaptive materials, setting the stage for the responsive architectures discussed in later sections. Fig. 3 summarizes the representative monomers employed in these systems, highlighting their key properties and relevance to ABC star design.

A pivotal advance came in 2012 with the systematic integration of anionic polymerization and click chemistry by Hanisch *et al.*^11^ Using a DPE–alkyne linker, they synthesized alkyne functionalized diblocks that were coupled to azide-terminated homopolymers *via* CuAAC. This modular approach tolerated bulky and functional moieties, including a perylene dye, and enabled further design of complex architectures.^76^

Several later studies adopted a 1:1:*x* design strategy, fixing two arms while varying the third, to investigate how asymmetry influences self-assembly. Antoine *et al.* used Steglich esterification to couple carboxy-terminated PI with an anionically synthesized PS–P2VP diblock,^50^ while Chernyy *et al.* employed fully anionic routes to prepare PS–PI–P2VP and PDMS–PI–PMMA stars with tunable asymmetry.^56,77^

A distinct subclass of anionic methodology is the iterative strategy developed by Hirao *et al.*,^78^ enabling stepwise construction of increasingly complex miktoarm stars through sequential coupling. While this route has produced ABC stars,^79,80^ its main contributions lie in showcasing elaborate architectures such as ABCDEFG stars^81^ and cross-linkable cores.^82^

Collectively, these studies underscore the architectural precision achievable *via* anionic polymerization. This remains the benchmark for defining ABC stars, forming the basis of most bulk and thin-film morphology investigations. However, as synthetic goals expanded toward functionality and broader monomer compatibility, alternative methods, particularly ROP, have gained prominence.

### 3.2 Opening the ring: biodegradable and amphiphilic stars

ROP developed in parallel with anionic methods as a versatile route for incorporating cyclic, biodegradable monomers. Unlike the precision-oriented focus of anionic systems, ROP marked a shift toward application-driven architectures emphasizing biodegradability and amphiphilicity. This stems from the fact that many cyclic esters and carbonates used in ROP yield hydrolytically cleavable backbones, enabling degradation under physiological conditions.^83^

**Fig. 4:**
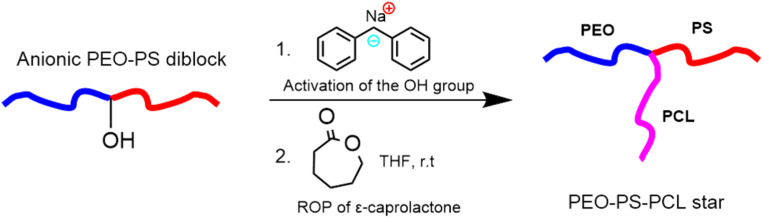
Synthetic pathway for a PS–PEO–PCL star, combining anionic and ring opening polymerization. Adapted from Lambert *et al.*^10^

Lambert *et al.* introduced the first ROP-based ABC star, incorporating hydrophilic PEO and biodegradable PCL segments into a PS–PEO–PCL star from a PS–PEO diblock bearing a *tert*-butyldimethylsilyl-protected hydroxyl group.^10^ Upon deprotection, the hydroxyl initiated ε-CL polymerization, as shown in Fig. 4. Follow-up studies replaced PEO with PMMA and introduced poly(l-lactide) (LLA) *via* Sn(Oct)_2_-catalyzed ROP, demonstrating compatibility with biodegradable monomers outside anionic conditions.^84–86^ Wang *et al.* later revisited the PS–PEO–PCL system *via* a shorter route, though broad dispersities persisted.^87^

A wide variety of monomers compatible with ROP have since been employed in ABC stars, enabling new functionalities and broadening biomedical relevance. PEO remains the most common hydrophilic block, frequently paired with biodegradable segments such as PCL, PLLA or poly(d,l-lactide) (PDLLA).^88–91^ Peptide-based systems have also emerged, incorporating poly(β-benzyl-l-aspartate) (PBLA) for biodegradability^92^ or redox-sensitive poly(l-lysine) (PLL) for responsive delivery systems.^93^ Other entries include fully ROP based stars containing functionalizable poly(β-benzylmalolactonate) (PBMA),^94^ and epoxide-based monomers which impart hydrophilicity and pH-responsiveness.^95,96^

**Fig. 5:**
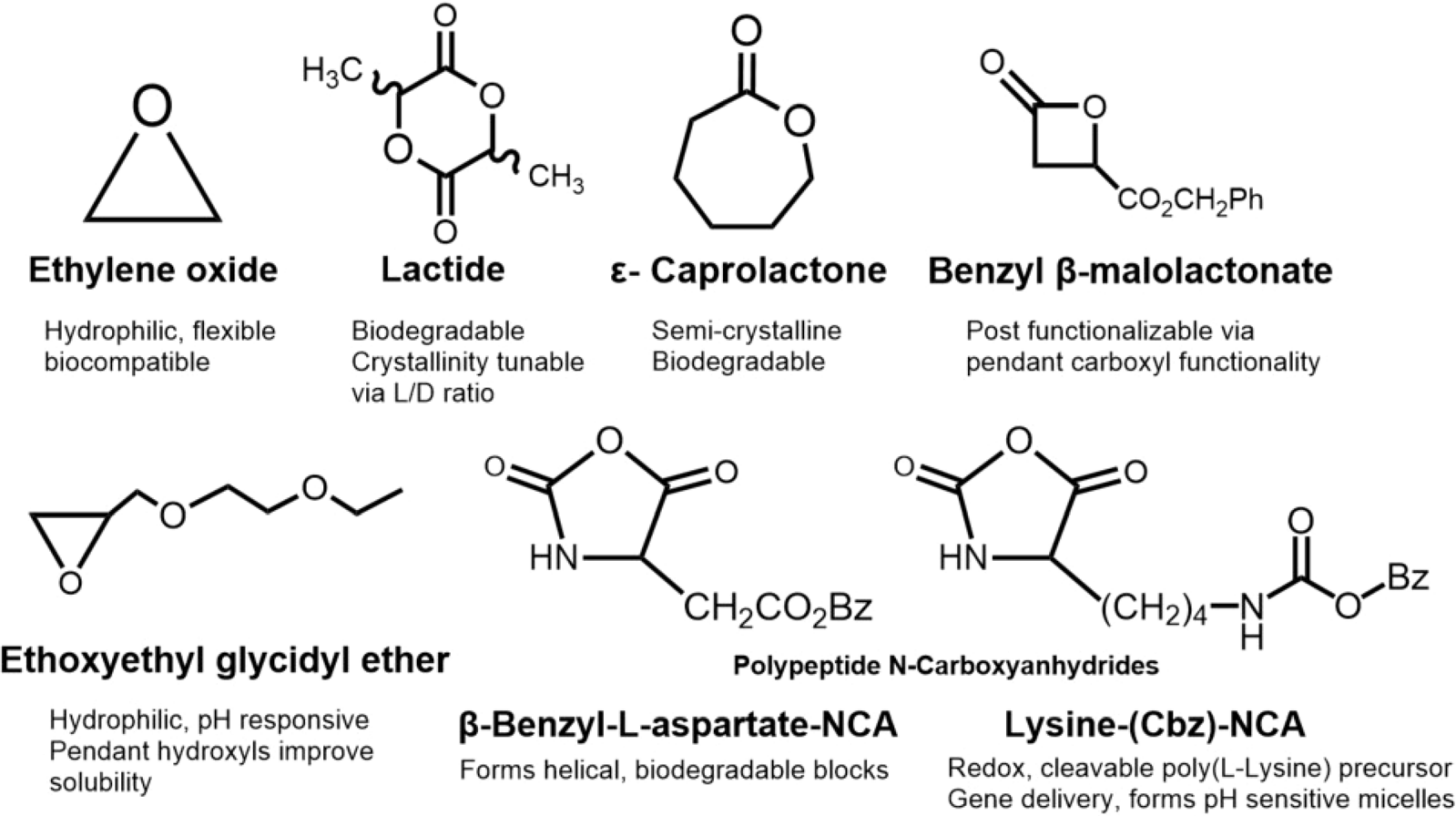
Common monomers incorporated into ABC stars *via* ROP, including biodegradable polyesters, hydrophilic epoxides, and functional polypeptide precursors.

Recent efforts also highlight the rise of metal-free polymerizations, using organocatalysts or enzymatic initiators to enhance biocompatibility, particularly for drug delivery applications where metal residues are detrimental.^93,97^ A visual summary of key ROP monomers and their properties is presented in Fig. 5.

These developments demonstrate how ROP-based strategies have shifted ABC star synthesis toward application-driven design. By enabling the incorporation of degradable, hydrophilic, and biofunctional segments under mild conditions, ROP provides synthetic flexibility well suited for responsive materials and biomedical use. However, its reliance on cyclic monomers and limited compatibility with complex end-group modifications restrict its architectural scope. To overcome these limitations, especially in the synthesis of modular and stimuli-responsive systems, RDRP has emerged as a dominant strategy.

### 3.3 Reversible deactivation radical polymerization: expanding the synthetic Frontier

The introduction of RDRP methods, such as ATRP, RAFT, NMP, and their derivatives, marked a turning point in ABC star synthesis. These techniques significantly broadened the accessible monomer range and enabled polymerization under mild, aqueous, or orthogonal conditions, at the cost of increased dispersity. While this limited structural resolution in some morphological studies,^98^ it was often acceptable, or even beneficial, in application-focused systems.^99^

The first hybrid synthesis was reported by Huang *et al.*, who combined anionic and photoinitiated radical polymerization to prepare PS–PEO–PMMA stars using photoactive PEO macroinitiators.^100,101^ A follow-up by Lu *et al.* replaced MMA with methacrylic acid (MA), introducing a pH responsive segment.^102^

Feng *et al.* later introduced the first ABC stars composed entirely of non-anionic arms by combining cationic ring-opening polymerization (CROP) with ATRP. This enabled the incorporation of flexible polyethers such as poly(1,3-dioxepane) (PDOP) and poly(tetrahydrofuran) (PTHF), which are incompatible with anionic methods.^103^ The same authors reported the first RAFT-based stars by linking PS–RAFT chains through a maleic anhydride core, followed by polymerization of PMA or PNIPAM and coupling of PEO *via* esterification.^104^ These strategies introduced both RAFT and thermoresponsive PNIPAM into ABC star synthesis. Later studies extended these approaches to include combinations such as PTHF–PDOP–PMMA and PEO–PS–PLLA, integrating RAFT, CROP, and ROP techniques.^105,106^

**Fig. 6:**
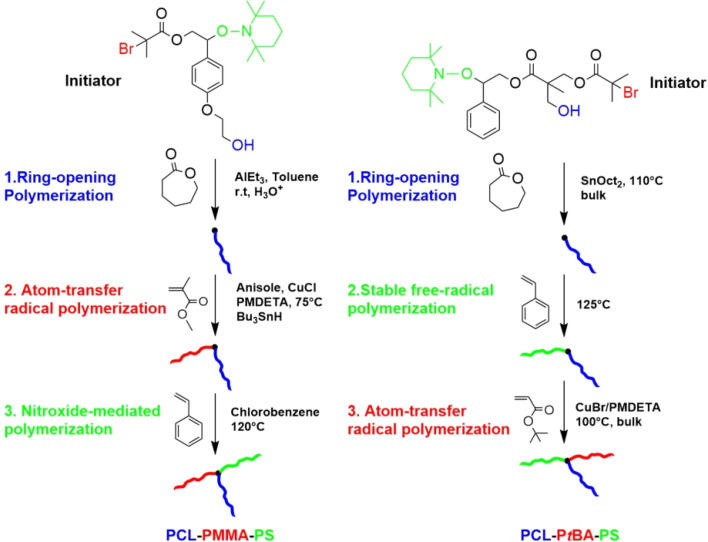
Schematic representation of the first-reported fully core-first syntheses, He *et al.*^107^ (left), Tunca *et al.*^108^ (right). The different polymerization mechanisms are color coded as follows: blue = ROP, green = NMP/FRP, red = ATRP.

Shortly after these developments, fully core-first strategies were reported using trifunctional initiators bearing orthogonal sites for ROP, ATRP, NMP and stable free radical polymerizations. He *et al.* synthesized PCL–PMMA–PS stars from initiators containing hydroxy, bromoisobutyrate, and alkoxyamine groups, though the NMP step showed limited initiation efficiency.^107^ Around the same time, Tunca *et al.* developed a similar PCL–PS–P*t*BA system using a core compatible with Sn(Oct)_2_-catalyzed ROP, Stable Free Radical Polymerization, and ATRP, achieving higher conversions but broader molecular weight distributions.^108^ A comparison of these strategies is shown in Fig. 6.

**Fig. 7:**
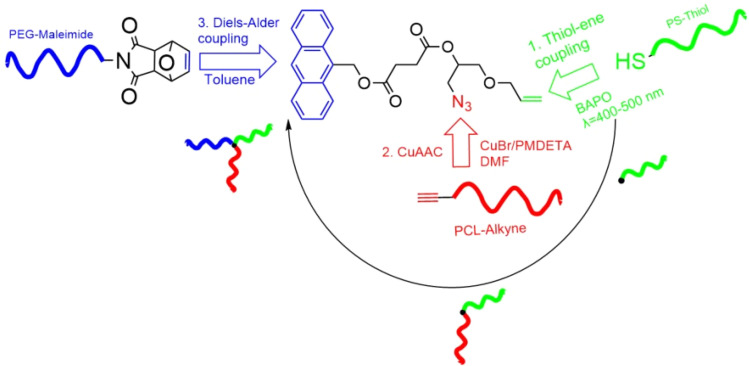
Modular approach to the synthesis of PS–PCL–PEO ABC star by Iskin *et al. via* three orthogonal “click” reactions. Adapted from ref. 12 with permission.

Tunca's group was the first to integrate RDRP and click chemistry in ABC star synthesis, modifying their previously reported core (Fig. 6, right) with an alkyne for CuAAC coupling of the third arm, yielding PS–PMMA–PEO and PS–PMMA–P*t*BA stars.^59^ Follow-up studies introduced DA^62^ and thiol–ene click reactions,^12,61^ using cores functionalized with anthracene, allyl, and hydroxyl groups. This progression culminated in a fully modular triple-click strategy: a core bearing azide, allyl, and anthracene moieties was reacted with alkyne–PCL, thiol–PS, and maleimide–PEO *via* CuAAC, thiol–ene, and DA chemistry, respectively, as shown in Fig. 7.^63^ Gunay *et al.* later extended this modular design to PEO–PCL–PS and PEO–PCL–poly(*N*-butyl oxanobornene imide) stars, introducing a photolabile block suitable for light-triggered degradation.^64^ Numerous related strategies have since appeared, most commonly combining CuAAC with ATRP, RAFT, or ROP.^109–113^

Dong *et al.* reported the first fully ATRP-based ABC stars in 2009, synthesizing PMMA-PEO-PS and pH-responsive PMAA-PEO-PS *via* sequential ATRP using a styrenic-terminated PEO junction.^114^ A related one-pot approach by Alizadeh *et al.* combined CuAAC and Single Electron Transfer Living Radical Polymerization (SET-LRP) to yield PEO–P*t*BA–PCL stars, later converted to PEO–PAA–PCL *via* selective acidolysis.^115^

Li *et al.* expanded modular strategies by applying the Passerini three-component reaction to install both an ATRP initiating site and an alkyne onto PEO, enabling PNIPAM–PEO–PS and P*t*BA–PEO–PS stars *via* ATRP, SET-LRP, and CuAAC in either sequential or one-pot setups.^116^ This offered a streamlined alternative to more complex core designs.

RDRP techniques enabled broad diversification of monomers and coupling strategies in ABC star synthesis. While often associated with higher dispersities, these methods allowed access to polar, protic, and charged blocks previously incompatible with ionic polymerizations. These features made it possible to explore systems that change morphology in response to pH, temperature, or light, expanding the functional scope beyond static, composition driven self-assembly. As a result, amphiphilic and stimuli-responsive ABC stars became a logical focus, both due to their synthetic accessibility through RDRP and their relevance to dynamic behavior in solution.

Among the earliest examples, Feng's incorporation of PNIPAM into ABC stars *via* RAFT^104^ marked a shift toward stimuli-responsive architectures. These frequently paired thermoresponsive PNIPAM with blocks sensitive to pH, such as poly(2-(diethylamino)ethyl methacrylate) (PDEA) or its isopropyl derivative (PDPA), poly(l-lysine) (PLys), or lightresponsive poly(*o*-nitrobenzyl methacrylate) (PNBM). Additional variants included zwitterionic poly(2-methacryloyloxyethyl phosphorylcholine) and redox-responsive poly(acryloylthiolactone) (PATL). The modularity of RDRP and click strategies enabled rapid growth of this subclass, with most systems designed for multi-responsive micellization in aqueous media.

**Fig. 8:**
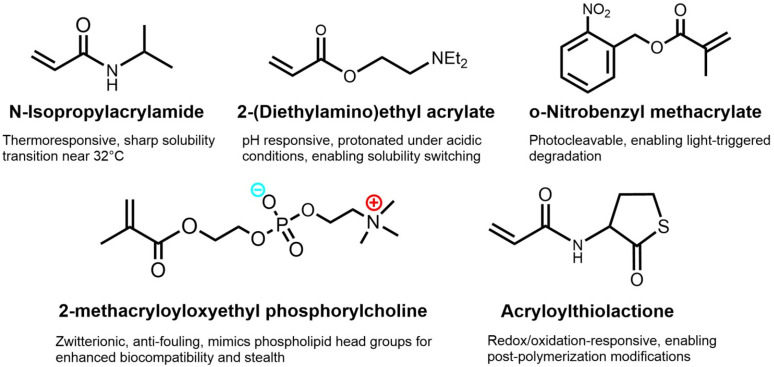
Monomers commonly used in the synthesis of responsive ABC stars.

Selected examples are summarized in Table 3, and the monomers are shown in Fig. 8.

**Table 3:** Representative responsive ABC stars grouped by stimulus type

	Type of responsiveness		
	Thermal	pH	Light/other
Relevant blocks	PNIPAM	PLys, PDEA, PAA	PNBM, PATL, PDPA
Synthesis	RAFT, ATRP, CuAAC	ATRP, ROP, CuAAC	ATRP, CuAAC, thiol–ene
References	104 and 117–119	120–124	125–127

**Fig. 9:**
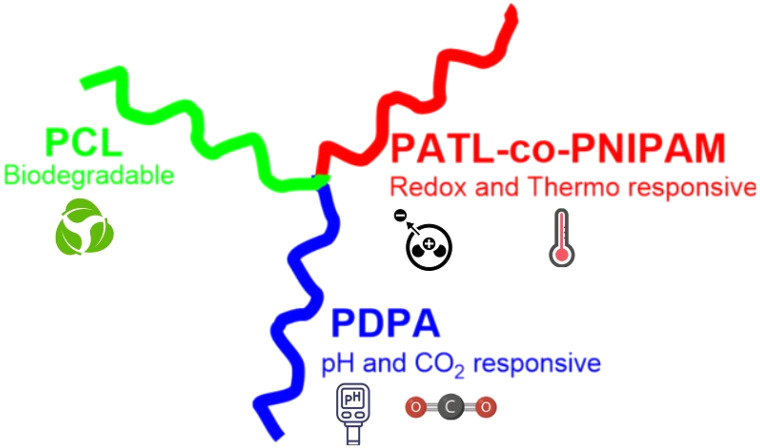
Schematic representation of the penta-responsive ABC star by Zhao *et al.*^128^

A culmination of these strategies was demonstrated by Zhao *et al.*, who synthesized a PDPA–(PATL-*co*-NIPAM)–PCL ABC star *via* a modular combination of RAFT, ATRP, and ROP polymerizations.^128^ The resulting architecture exhibited responses to temperature, pH, CO_2_, redox, and oxidative stimuli. Additionally, reversible hydrogen bonding at the Y-shaped junction contributed to its dynamic solution behavior. Responsiveness was evaluated through cloud point measurements, Dynamic Light Scattering (DLS), and Transmission Electron Microscopy (TEM), revealing diverse morphologies under different conditions. The structure and responsiveness of this system are illustrated in Fig. 9.

### 3.4 Unconventional designs and synthetic innovation

While most ABC star systems fall into recognizable synthetic categories, a subset of entries stand out for their conceptual novelty, employing unconventional cores, topologies, polymerization methods, or coupling strategies to access new functions or assembly behaviors.

One early deviation from standard designs was the use of bio-derived cores. Wei and Huang introduced a lysine-based trifunctional junction in a PI–PS–PEO star, marking the first naturally sourced scaffold in ABC synthesis.^129^ This approach evolved into broader bioinspired strategies, incorporating polypeptides and peptide mimics *via N*-carboxyanhydride polymerization, often imparting secondary structure, optical activity, or gene delivery functionality.^130–134^

**Fig. 10:**
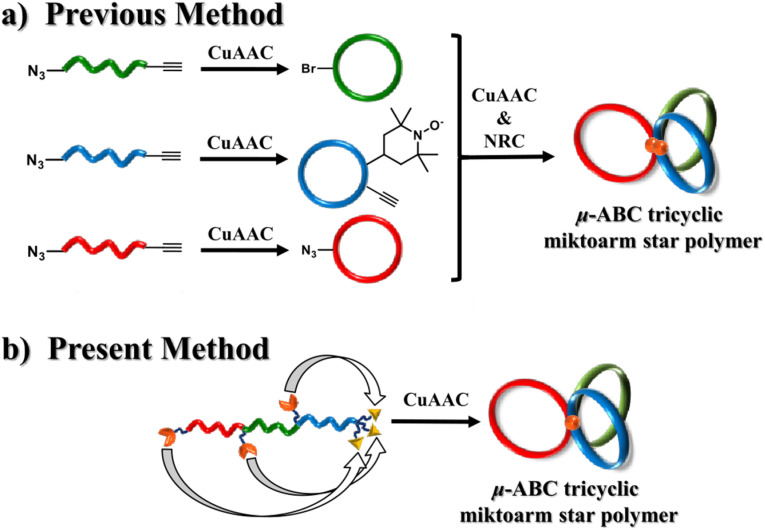
Contrast between approaches by Jie *et al.* (a)^135^ and by Shingu *et al.* (b)^60^ for obtaining tricyclic ABC stars. Reproduced from Shingu *et al.*^60^

Topological innovation was introduced by Jia *et al.*, who synthesized the first fully cyclic ABC star by coupling three individual cyclic polymers, PS, PMA, and P*t*BA, *via* CuAAC and nitrogen radical coupling reactions.^135^ Shingu *et al.* later streamlined the strategy by employing slow-addition CuAAC on a linear triblock precursor, enabling simultaneous intramolecular cyclization of all three segments in a single step,^60^ the respective approaches shown in Fig. 10.

Light has served as both a synthetic handle and a functional trigger in ABC star design. Azobenzene-containing blocks enabled photo-responsive conformational switching, particularly when combined with rigid structural elements.^136,137^ Expanding on this theme, Eibel *et al.* developed a fully light-controlled core-first approach using wavelength-selective free radical polymerization to sequentially grow three methacrylate-based arms from a chromophore bearing core. This strategy offered metal-free control but exhibited relatively high dispersities.^138^

Some efforts have prioritized synthetic modularity over targeted function. Ge *et al.* employed reversible complexation-mediated polymerization with end-group transformations to control sequential arm growth,^139^ while Nagashima *et al.* used a silver-catalyzed, CO_2_ mediated alkynoate coupling, followed by amino–yne addition to complete the star.^140^

Finally, polymerization-induced self-assembly (PISA) has been adapted for ABC stars. Li *et al.* and Zhao *et al.* used RAFT polymerization to simultaneously grow the third arm and induce nanoparticle formation *in situ*, providing a scalable route to solution-phase star formation in selective solvents.^141,142^

While no single synthetic approach dominates ABC star development, clear trade-offs have emerged. Anionic and ROP based methods offer high structural precision and remain central to morphology focused studies. RDRP techniques, often combined with orthogonal linking strategies, expand functional scope and modularity, particularly for responsive or application driven systems. Table 4 presents a selection of representative ABC stars, highlighting how advances in synthetic strategy have enabled increasingly functional and complex architectures. These developments underpin the application-oriented examples discussed in the following section.

**Table 4:** Chronological summary of selected ABC star polymers, highlighting key synthetic approaches, linking strategies, and notable functional features

Year	Technique	Linking strategy	ABC star	Notable features	Ref.
1991	Anionic	DPE	PDMS–PS–P*t*BMA	First reported ABC star	30
1997	Anionic, CTP	Radical coupling	PEO–PS–PMMA	First radical polymerization	100
1997	Anionic, ROP	DPE	PEO–PS–PCL	First use of ROP	10
2001	CROP, ATRP	Fully “core-first”	PTHF–PDOP–PS	Fully non-anionic approach	103
2002	RAFT	Esterification	PEO–PS–PMA	Introduction of RAFT and responsive PNIPAM segment	104
2006	ATRP, NMP	CuAAC	PS–PMMA–PEO	First use of “click chemistry"	59
			PS–PMMA–P*t*BA		
2008	Anionic, ROP	Nucleophilic substitution	PEO–P2VP–PCL	Responsive micelles	143
2009	ROP, NMP	Esterification	PEO–PLA–PS	Encapsulation of anti-cancer drug, paclitaxel	144
			PEO–PLLA–PDLLA		
2011	RAFT, ROP	CuAAC, thiolene, DA	PEO–PS–PCL	Three orthogonal click reactions	63
2012	ATRP, SET-LRP	CuAAC	PS–PMA–P*t*BA	Tricyclic structure	135
2017	FRP	Fully “core-first”	PMMA–PBMA–PBzMA	Light-triggered orthogonal polymerization	138
2020	FRP, ROP, ATRP	Fully “core-first”	(PATL-*co*-PNIPAM)–PCL–PDPA	Pentaresponsive system	128
2023	ROP, ATRP, RAFT	Fully “core-first”	PCL–PMMA–P4VP	Responsive PISA	142

### 3.5 Applications of ABC stars

ABC star polymers have been proposed for applications such as drug delivery and lithography due to their unique ability to combine incompatible segments within a single, well-defined architecture. Their responsive arms offer selective swelling or collapse in aqueous media, relevant for delivery systems,^18^ while microphase-separated domains offer potential for templated etching in lithography.^145^ Despite these conceptual advantages, few designs have been evaluated in practice. The synthetic complexity, multistep procedures, and limited scalability have slowed translation into real-world applications.

Among proposed applications, drug and gene delivery has seen the most extensive exploration in ABC star systems, by virtue of integrating chemically distinct segments into single molecule capable of micellar self-assembly. The pioneering example was the PEO–PCL–P2VP system by Butsele *et al.*,^88^ exhibiting a pH-responsive micellar behavior.^143^ This system demonstrated tumor-targeted drug delivery using Nile Red as a model drug.^146^ Other studies also used model hydrophobic dyes for mechanistic evaluation.^112,127^ Subsequent studies expanded to therapeutic cargos, anticancer drugs such as paclitaxel,^144^ doxorubicin^92^ or Coenzym Q10 (ref. 147) achieved exceptional loading with sustained release. A notable co-delivery design by Liu *et al.* employed a PEO–PCL–PLL star, where the hydrophobic core encapsulated paclitaxel while the cationic PLL arm complexed plasmid DNA, enabling simultaneous gene and drug delivery.^148^ Soliman *et al.* designed a fluorescent system enabling real-time tracking, that achieved 25 000-fold increase in aqueous solubility of curcumin.^149^ Alizadeh *et al.* demonstrated pH-dependent naproxen release in a PEO–PAA–PCL star: suppressed in gastric conditions, but triggered under intestinal pH.^115^ These are summarized in Table 5.

**Table 5:** ABC stars in drug delivery, adapted from ref. 18

ABC star	Stimulus	Cargo	Notable features	Ref.
PEO, PCL, P2VP	pH	Nile red	Minimal toxicity	146
PEO, PLLA, PDLA	N/A	Paclitaxel	High anticancer drug loading	144
PEO, PCL, TPPBr	N/A	CoQ10	Exceptional loading capacity (≥60 wt%)	147
PEO, PCL, PLL	N/A	Paclitaxel, DNA	Co-delivery capabilities	148
PEO, PCL, PBLA	N/A	Doxorubicin	Non-toxic micelles with anticancer activity	92
PEO, PCL, TIF	N/A	Curcumin	25 000 fold increase in aqueous solubility fluorescence tracking	149
PEO, PAA, PCL	pH	Naproxen	pH dependent release, low at gastric pH, high at intestinal pH	115
PEO, PNBM, PNIPAM	UV, pH, Δ*T*	Nile red	Multi-stimuli responsive release mechanism	127

The few non-biomedical exceptions demonstrate promising advantages. In flame retardancy, Qiu *et al.* investigated polyphosphoester (PPE) based stars, achieving a 23% increase in char yield and 9% reduction in peak heat release rate compared to the linear analogue, due to dense, multibranched structure enhancing phosphorus content.^150^ Similarly, in solid-state electrolytes, Su *et al.* used PS–PLA–PEO stars in PEO-based electrolytes, achieving a 3.6× increase in tensile strength (from 2.4 to 8.7 MPa) and 2× higher ionic conductivity (0.456 mS cm^−1^ at 70 °C *vs.* 0.2 mS cm^−1^ for pure PEO) by leveraging microphase separated domains to decouple mechanical and ion-transport properties,^5^ effectively mitigating the classical tradeoff between conductivity and mechanical robustness observed in linear or heavily crosslinked systems.^151,152^

Other fields remain largely unexplored. For instance, BCPs have been explored in nanolitography due to their ability to form ordered patterns with sub-100 nm feature sizes through self-assembly.^153^ While linear ABC triblocks achieve square arrays^154^ and hole patterns,^155^ no lithographic applications of ABC stars exist up to date. This gap stems from synthetic challenges: long-range order in thin films demands tight control over architectural parameters, including arm symmetry^77,156^ and molecular dispersity. While some studies suggest that high dispersity can increase defectivity and disrupt domain periodicity in block copolymer thin films,^98,145,153^ the precise impact remains insufficiently understood, especially for complex architectures like ABC stars. Their architectural asymmetry could theoretically enable novel surface patterns, but this potential remains locked behind synthetic barriers for the time being.

## 4 The morphological behavior of ABC stars

### 4.1 Bulk and thin-film morphologies

Morphology has been a defining focus of ABC star studies from the beginning. The convergence of three chemically distinct arms at a single junction inherently introduces packing frustration and interfacial asymmetry, leading to morphologies inaccessible to linear triblocks. This architecture not only affects phase behavior but also imposes unique spatial constraints on the molecular scale. In contrast to linear systems, where block junctions are distributed across intermaterial dividing surfaces, ABC stars have been shown to confine their junction points to lines where all three domains meet.^157,158^ Early studies treated self-assembly as an indirect probe of these architectural effects. Representative cases include cylindrical domains in PS–PI–PB stars despite lamellae-favoring volume fractions,^159^ unassigned tricontinuous structures in PS–PDMS–P*t*BMA,^160^ and two-dimensional tilings proposed in PS–PI–PMMA systems.^157,161^ Broader compositional series suggested cylinder-to-lamellae transitions, though junction localization remained unclear.^66,67^ Notably, Hückstädt *et al.* identified a core–shell double gyroid in PS–PB–P2VP stars as early as 2000,^162^ and Yamauchi *et al.* later used 3D electron tomography to resolve non-classical, curved multi-cylinder packings in PS–PI–PDMS stars, structures attributed to mutual repulsion between the arms.^163^ These studies foreshadowed the structural complexity only fully appreciated in the following decade.

The full structural complexity of ABC stars became accessible through advances in imaging and scattering techniques, including Small- and Wide-Angle X-ray Scattering (SAXS, WAXS), Grazing-Incidence Small-Angle X-ray Scattering (GISAXS) for surface morphology, Transmission and Scanning Electron Microscopy (TEM, SEM), and Atomic Force Microscopy (AFM). These complementary methods, summarized in Table 6, enabled direct visualization and quantification of novel assemblies unique to ABC stars.

**Table 6:** Summary of characterization techniques used for morphological assignment in ABC star polymers

Technique	Specialty	Applicable in	Key contributions	Ref.
SAXS	Nanostructure spacing (1–100 nm), phase contrast^164^	Solution, bulk thin films	Domain spacing, characteristic length	165 and 166
WAXS	Crystalline packing, short-range order^167^	Bulk, semicrystalline films	Detecting crystalline domains (PS, PCL *etc.*)	131
GISAXS	Lateral structure at interfaces, periodicity^168^	Thin films	Surface microphase separation, lateral periodicity	55, 75 and 169
TEM	High resolution morphology, contrast *via* staining^170^	Thin films, freeze dried micelles	Direct imaging of the morphology	157, 171 and 172
SEM	Surface topography^173^	Thin films	Surface domain mapping, less common	174 and 175
AFM	Topography and phase imaging^176^	Thin films	Surface morphology, phase contrast	175, 177 and 178
DLS	Hydrodynamic radius, size distribution^179^	Solution	Monitoring micelle formation and stimuli induced shifts	126, 180 and 181
*ζ*-Potential	Surface charge estimation^182^	Solution	Tracking pH or ionic response of micelles	115 and 143

Theoretical work has shown that even minor additions of homopolymers can significantly influence the self-assembly of ABC stars, modulating domain geometry, symmetry, and junction localization.^183^ Experimentally, such blending has been used to either stabilize or disrupt ordered morphologies. For example, selective homopolymer addition was found to straighten distorted domains and increase grain size,^184^ stabilize otherwise fragile tiling patterns like (4.8.8),^177^ or suppress competing morphologies in thin films.^169^ In other cases, blending perturbed domain packing and gave rise to new phases such as core–shell gyroids.^185^ These studies demonstrate how a relatively simple homopolymer incorporation can serve as a powerful tool to tune ABC star morphology across both bulk and confined systems.

The 1:1:*x* compositional approach, fixing two block ratios while varying the third, has become a powerful method to explore how asymmetry influences domain geometry. Takano *et al.* applied this strategy to PI–PS–P2VP (ISP) stars, revealing tiling transitions from honeycomb (6^3^) to square–octagon (4.8.8) and dodecagonal (4.6.12) phases as the P2VP fraction increased,^186^ consistent with predictions by Gemma *et al.*^158^ Hayashida *et al.* confirmed these morphologies using microbeam SAXS, identifying additional patterns such as (6.6.6), (3.3.4.3.4), and distorted variants caused by grain orientation and frustration.^165^

**Fig. 11:**
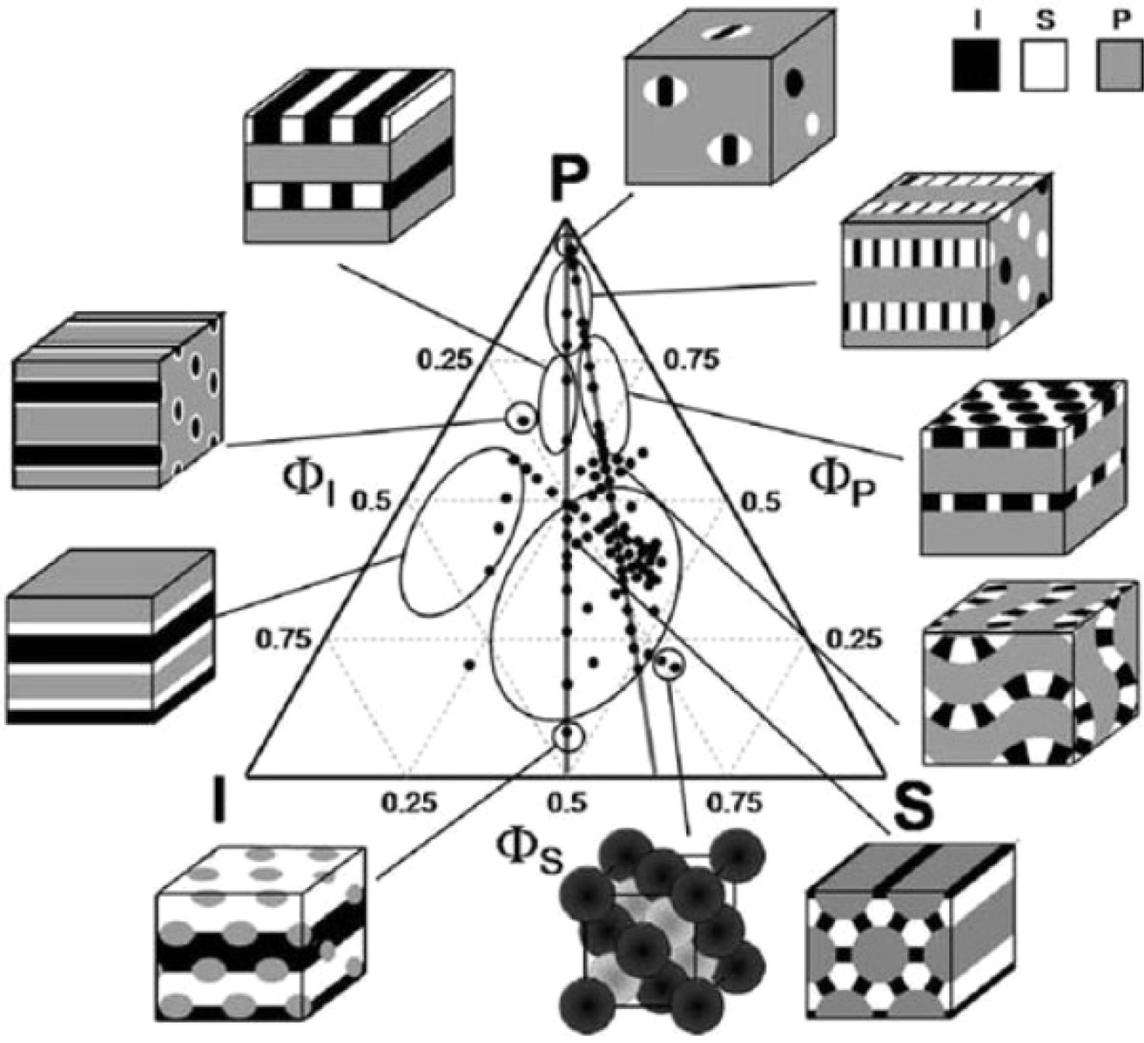
Ternary phase diagram of the ISP star. Reproduced with permission from ref. 16.

Subsequent studies extended this to homopolymer blends, uncovering rare tilings like [5.3,5.3,8] and [4.5,6,9], with transitions rationalized by average coordination number rules.^187^ A full ISP phase diagram was later mapped across 0.2 ≤ *x* ≤ 10 (shown in Fig. 11), revealing transitions from spherical to cylindrical and hierarchical structures, with increasing disorder at high asymmetry.^172^ Hayashida *et al.* also characterized nested structures such as lamellae-in-cylinder and lamellae-in-sphere, shaped by interfacial tension and junction confinement.^156^

Matsushita *et al.* unified these results into a ternary morphological map incorporating both ISP and SBV (PB-substituted) stars, where similar topologies, quasicrystalline tilings, ZnS-type networks, and hybrid morphologies, were observed despite slight differences in flexibility.^16,17^ Together, these studies, including thin-film variants where hydrogen bonding or solvent annealing enabled large-unit-cell and dielectric-dependent (4.6.12) tilings,^188,189^ as well as later compositional extensions confirming frustration-driven transitions *via* SCFT,^56^ form the most comprehensive structural investigation of any ABC star system to date.

The 1:1:*x* strategy has also been extended to chemically distinct systems. Chernyy *et al.* studied PDMS–PI–PMMA (DIM) stars, observing transitions from (6.6.6) tilings to alternating lamellae and hierarchical cylinders with increasing PMMA content.^77^ In thin films, SEM and etching revealed morphologies such as square arrays of PDMS nanodots and empty-core cylinders. Ariaee *et al.* investigated a homologous ISM system (PI–PS–PMMA), reporting bulk lamellar-to-core–shell transitions and thickness-dependent symmetry shifts in films, culminating in square-packed vertical cylinders at high PMMA content.^175^

More recently, Moschovas *et al.* reported a PS-PI-PB star forming a long-range ordered *I*4_1_32 cubic phase, interpreted as alternating gyroid networks of two interpenetrating polydiene domains within a PS matrix.^166^ The junction-imposed connectivity distinguished this from conventional double gyroids, highlighting how miktoarm topology can stabilize topologically complex bulk phases. This marks a conceptual high point in bulk ordering, beyond which, self-assembly shifts toward kinetic control, micellar architecture, and solvent-directed organization.

### 4.2 Solution self-assembly

ABC star polymers provide a compelling platform for multicompartment micelles, which combine incompatible functionalities within a single nanostructure and enable applications such as simultaneous delivery, catalysis, or orthogonal reactivity. Early demonstrations relied on chemically distinct arms to drive internal segregation. Li *et al.* synthesized stars composed of PEE, PEO and fluorinated blocks using anionic polymerization and acyl chloride–alcohol coupling, achieving clear domain separation due to the immiscibility of the fluorinated segment.^190^ Saito *et al.* similarly reported PEE–PEO–PMCL stars incorporating a degradable polyester block *via* AlEt_3_-catalyzed ROP,^191^ while later optimizations by Abouelmagd and Liu improved solubility and contrast in fluoropolymer-containing stars.^174,192^

**Fig. 12:**
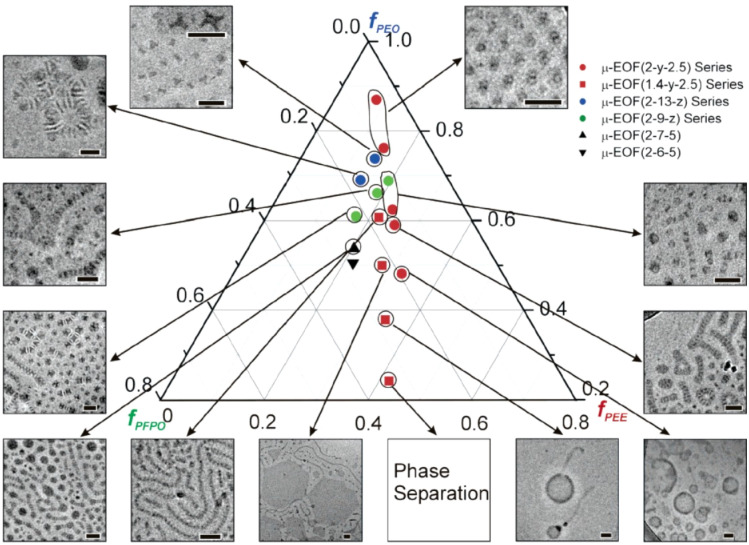
Ternary phase diagram of an EOF star system. Reprinted with permission from ref. 180.

Despite these advances, early self-assembly studies were limited by weak segregation and low contrast. Castelletto *et al.* examined methacrylate-based stars in aqueous solution and observed no distinct microphase separation, instead modeling them as swollen Gaussian coils.^193^ Nonetheless, subtle effects of block sequence and temperature hinted at architecture governed organization even in the disordered state. Clearer micellar morphologies emerged with the use of compositionally incompatible or high-contrast blocks, which enhanced segregation and internal structure. A hallmark of ABC stars in aqueous media is the formation of multicompartment micelles with segmented, raspberry-like, or wormlike cores. Saito's EOC system exhibited transitions from spheres to worms to disks across a composition gradient,^191^ while Li's EOF stars formed hamburger, raspberry, and wormlike micelles with distinct internal compartmentalization, yielding a ternary phase diagram shown in Fig. 12.^180^

**Fig. 13:**
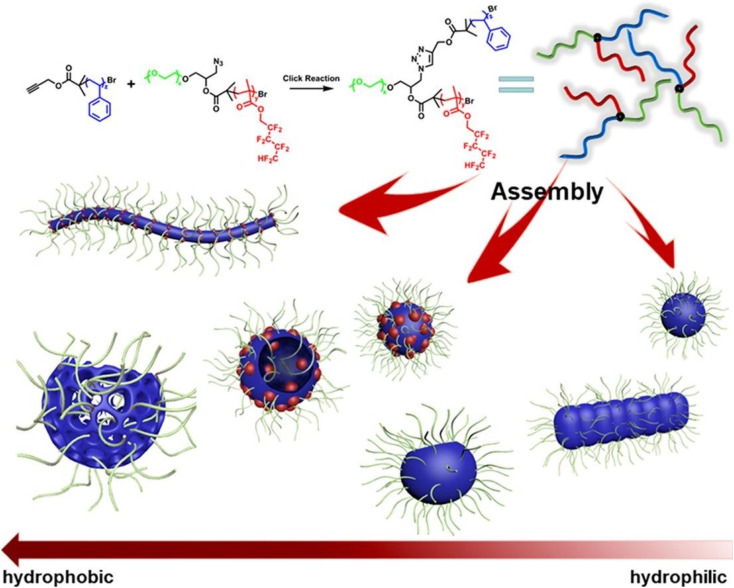
Representative micelles formed by an ABC star containing a perfluorinated segment, illustrating composition-dependent self-assembly into patchy spheres, segmented worms, vesicles, stomatocytes, and raspberry-like structures. Adapted with permission from ref. 174.

Subsequent studies expanded the compositional space of multicompartment micelles using strongly immiscible or fluorinated block combinations. Liu *et al.* systematically varied PEO, PS, and fluorinated blocks to access patchy spheres, segmented worms, vesicles, and perforated stomatocytes, shown in Fig. 13, with cryo-TEM and SEM confirming internal phase segregation across ternary composition maps.^174,194^ Li *et al.* further demonstrated multicompartment nanoparticles with raspberry-like surfaces and partially segregated PS cores, synthesized *via* seeded RAFT polymerization.^141^

### 4.3 Stimuli-responsive solution self-assembly

**Fig. 14:**
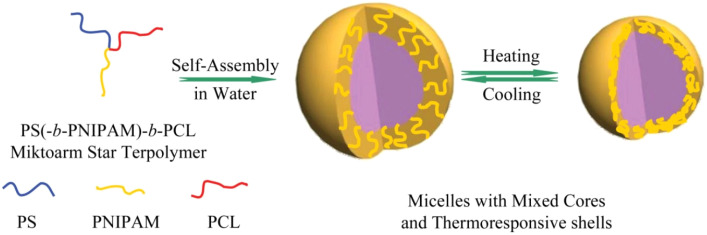
Schematic depiction of a thermally-induced corona collapse in a PS-PNIPAM-PCL ABC star. Adapted with permission from ref. 117.

Beyond compositionally driven self-assembly, several ABC star systems have been engineered to respond to external stimuli, physical or chemical inputs that modulate solubility, charge, or hydration of individual blocks, resulting in dynamic morphological transitions. Zhang *et al.* reported PNIPAM-containing stars that formed spherical micelles with thermoresponsive coronas; above the Lower Critical Solution Temperature (LCST), the PNIPAM chains collapsed, leading to denser coronas and reduced hydration (Fig. 14).^117^ In a separate study, double-hydrophilic PEO–PNIPAM–PDEA stars exhibited “schizophrenic” micellization, in which the core–corona structure reversed in response to temperature or pH changes.^120^ This behavior arose from the environment-dependent solubility of PNIPAM and PDEA, allowing different arms to drive aggregation under distinct conditions. Yuan and Wang observed transitions from spherical micelles to nanorods in MPEO–PCL–PPE stars upon heating above the PPE cloud point.^181^

**Fig. 15:**
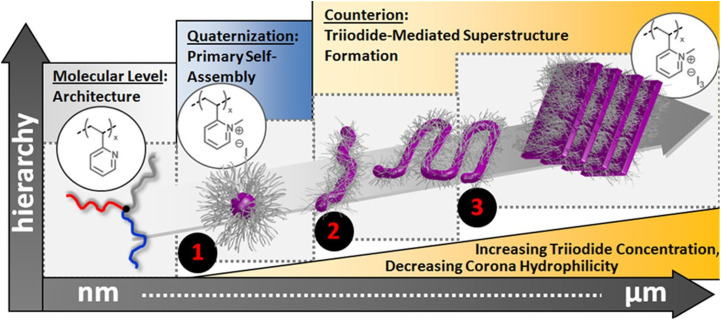
Mechanism for a formation of “superstructure” in PB–P*t*BMA–P2VP ABC star, triggered by complexation of P2VP with methyl iodide. Adapted from ref. 195.

Hanisch *et al.* showed a counterion-induced transformation in miktoarm stars containing P2VP, where spherical micelles hierarchically assembled into lamellar “woodlouse” morphologies upon complexation with methyl iodide. This process was driven by electrostatic interactions and corona bridging (intermicellar association *via* the charged P2VP segments) between neighboring micelles, leading to the formation of wormlike intermediates and eventually stacked lamellae, as illustrated in Fig. 15.^76,195^

Nunns *et al.* used crystallization of the ferrocene block to drive anisotropic micelle growth, forming elongated structures with coronas that segregated into alternating domains due to phase separation between the solvated arms.^178^ CO_2_ exposure triggered vesicle-to-sheet transitions in PEO–PS–PDEA stars by protonating the tertiary amine groups of the PDEA block, rendering it hydrophilic and shifting interfacial curvature to favor lamellar morphologies.^125^ Xu *et al.* reported a PEO–PNIPAM–PNBM system forming nanosheets that reorganized into vesicles or patchy spheres in response to light or temperature stimuli. These transitions were driven by PNIPAM collapse above its LCST and photoinduced cleavage of nitrobenzyl groups in PNBM, increasing corona polarity and altering curvature.^123^

Unlike bulk systems, where morphology is governed by long-range packing frustration and interfacial curvature, solution-phase assemblies prioritize dynamic responsiveness and internal segregation within discrete nanostructures. The ABC star architecture enables access to both regimes, bridging static phase behavior with functional adaptability.

## 5 Conclusion and future outlook

Despite advances in synthetic methodology, ABC star polymers remain fundamentally constrained by their complexity and the limited availability of functionalized macromonomers and core molecules. Anionic polymerization offers unmatched precision but depends on a narrow set of initiators and end-functionalizable monomers, many of which are not commercially available. Even relatively simple intermediates often require multistep synthesis tailored to specific research goals. While RDRP and click-enabled methods offer greater modularity, the absence of a standardized set of polymer precursors with orthogonal end groups continues to limit broader deployment.

A central, unresolved challenge is the lack of a modular synthetic toolkit. Most ABC star architectures still rely on custom-synthesized arms and cores, designed for specific studies and rarely generalizable. Without a shared library of end-functionalized polymers and compatible junction-forming cores, reproducibility remains low and throughput limited. Until such a plug and play system emerges, ABC star synthesis will remain labor intensive and fragmented, hindering both exploratory research and scalable application. To overcome this, future work should prioritize: establishing libraries of end-functionalized polymer precursors (*e.g.*, alkyne-PS, azide-PEO, PLA-COOH) to enable modular assembly, as demonstrated for complex architectures like dye-conjugated stars^11^ and developing machine learning tools for retrosynthetic planning, extending beyond morphology prediction^196^ to forecast monomer compatibility and side reactions, reducing trial-and-error.

Recent advances in machine learning have introduced new possibilities for predicting and guiding ABC star polymer design prior to synthesis. Initially developed in the context of simpler linear BCPs, inverse design approaches have used neural networks to predict SAXS profiles and suggest candidate architectures with desired morphologies.^197–199^ Cui *et al.* extended this approach to ABC miktoarm stars, applying deep learning models to simulate self-assembly under solvent evaporation. Their models generated synthesis–field diagrams that linked dispersity, composition, and morphology, providing insight into how molecular features and processing conditions govern structural outcomes.^196^ These tools could be strategically directed toward resolving key bottlenecks, such as predicting orthogonal monomer reactivity or optimizing solvent systems for specific arm combinations.

Despite the architectural elegance and tunable properties of ABC star polymers, a key question remains: what justifies their complexity? These systems require extensive synthetic effort and comprehensive characterization, often taking months to produce and test a single composition. For ABC stars to transition from academic demonstrations to impactful materials, future research must focus not only on expanding morphological diversity but on linking structure to functional outcomes beyond drug delivery. This demands a paradigm shift toward application-driven synthesis: targeting domains where ABC topology offers advantage, as demonstrated in solid electrolytes (*e.g.*, enhanced ionic conductivity/mechanical balance^5^) or flame retardancy (*e.g.*, improved char yield^150^), rather than forcing complexity where linear polymers suffice. Co-designing materials with end-use specifications, using computational tools^196^ to identify architectures that meet application-specific requirements (*e.g.*, degradation profiles, thermal stability).

Without these shifts, ABC stars risk remaining isolated curiosities, rich in potential but confined to niche biomedicine. A collaborative ecosystem, shared libraries, computational screening, application-focused design, is essential to unlock their broader materials innovation potential.

## Abbreviations

AFM: Atomic Force Microscopy
ATRP: Atom Transfer Radical Polymerization
BCP: Block Co-Polymer
CROP: Cationic Ring-Opening Polymerization
CuAAC: Copper Catalyzed Azide–Alkyne Cycloaddition
DA: Diels–Alder
DLS: Dynamic Light Scattering
DPE: 1,1-Diphenylethylene
GISAXS: Grazing Incidence Small Angle X-ray Scattering
LCST: Lower Critical Solution Temperature
NMP: Nitroxide Mediated Polymerization
P2VP: Poly(2-vinylpyridine)
PAA: Poly(acrylic acid)
PATL: Poly(acryloylthiolactone)
PB: Poly(butadiene)
PBLA: Poly(benzyl-l-aspartate)
PDEA: Poly-2-(*N*,*N*-diethylamino)ethyl methacrylate
PDMS: Poly(dimethylsiloxane)
PDOP: Poly(1,3-dioxepane)
PDPA: Poly-2-(*N*,*N*-diisopropylamino)ethyl methacrylate
PEO: Poly(ethylene oxide)
PI: Poly(isoprene)
PISA: Polymerization Induced Self-Assembly
PLA: Poly(lactide) (l = PLLA, d,l = PDLLA)
PLys: Poly(l-lysine)
PMA: Poly(methacrylic acid)
PMMA: Poly(methyl methacrylate)
PNBM: Poly(*o*-nitrobenzyl methacrylate)
PNIPAM: Poly(*N*-isopropylacrylamide)
PS: Poly(styrene)
PTHF: Poly(tetrahydrofuran)
RAFT: Reversible Addition–Fragmentation Chain Transfer
RDRP: Reversible Deactivation Radical Polymerization
ROP: Ring Opening Polymerization
SAXS: Small Angle X-ray Scattering
SEM: Scanning Electron Microscopy
SET-LRP: Single Electron Transfer Living Radical Polymerization
TEM: Transmission Electron Microscopy
WAXS: Wide Angle X-ray Scattering

## Author contributions

The manuscript was written through the contributions of all authors. Kalina M.: writing – original draft preparation, conceptualization, literature search, investigation, visualisation, data curation. Nouri, B.: validation, writing – review and editing. Almdal, K.: funding acquisition, supervision, resources. All authors have given approval to the final version of the manuscript.

## Conflicts of interest

There are no conflicts to declare.

## Data Availability

No primary research results, software, or code have been included, and no new data were generated or analyzed as part of this review.
